# Home blood pressure monitoring and adherence in patients with hypertension on primary prevention treatment: a survey of 1026 patients in general medicine in the Auvergne region

**DOI:** 10.1186/s12875-022-01725-8

**Published:** 2022-05-26

**Authors:** Jéromine Trefond, Lucie Hermet, Céline Lambert, Hélène Vaillant-Roussel, Denis Pouchain, Thibault Ménini, Bruno Pereira, Philippe Vorilhon

**Affiliations:** 1 Département de Médecine Générale, Université Clermont Auvergne, UFR Médecine, 28 place Henri Dunant, 63000 Clermont-Ferrand, France; 2 Université Clermont Auvergne, UR ACCePPT, 63000 Clermont-Ferrand, France; 3 Biostatistics Unit, DRCI, CHU Clermont-Ferrand, 63000 Clermont-Ferrand, France; 4 Department of General Practice, Faculty of Medicine, François Rabelais University, BP 3223, 37032 Tours Cedex, France

**Keywords:** Hypertension, Home blood pressure monitoring, Adherence, Guidelines, General practice, Cross-sectional survey

## Abstract

**Background:**

Home blood pressure monitoring (HBPM) could improve blood pressure control through therapeutic adherence. The main objective of this study was to determine the link between HBPM used by hypertensive patients treated in primary care and their medication adherence.

**Methods:**

Cross-sectional comparative study conducted in the Auvergne region from June to November 2016. Patients were recruited by general practitioners (GPs) selected at random. Adherence was evaluated according to the Girerd score.

**Results:**

From a sample of eighty-two GPs including 1026 patients, 45% of patients reported owning an HBPM device. Among these, 18% knew the rule of 3 (3 measurements in the morning and 3 in the evening for 3 days) recommended by the French State Health Authority. There was no difference in adherence between patients using HBPM and those who did not. Patients with HBPM using the rule of 3 reported better adherence than patients without the device (*p* = 0.06), and those who did not perform self-measurements according to the rule of 3 (*p* = 0.01). Patients who used HBPM according to the rule of 3 were older (*p* = 0.006) and less smokers (*p* = 0.001) than the others. Their GPs were more often GP teachers (*p* < 0.001) who practiced in rural areas (*p* = 0.001).

**Conclusion:**

The statistical link between medication adherence and HBPM for patients who apply the rule of 3, emphasizes the importance of the GP educating the patient on the proper use of HBPM.

**Supplementary Information:**

The online version contains supplementary material available at 10.1186/s12875-022-01725-8.

## Background

Common guidelines recommend home blood pressure monitoring (HBPM) for the diagnosis and monitoring of hypertension [1–4]. HBPM has been shown to reduce blood pressure (BP) levels, especially when combined with co-interventions such as self-medication titration or lifestyle counselling by pharmacists or nurses [5]. Self-monitoring of BP in the home environment is generally appreciated by patients and improves the doctor–patient relationship [6]. Many studies have evaluated the impact of HBPM on adherence to treatment. The results are not consistent from one study to another, being sometimes in favour of an improvement, especially when combined with co-interventions, and sometimes with no effect [5–8].

Vrijens defined adherence to therapy as the way in which the patient takes the treatments as prescribed, with three quantifiable phases: initiation, implementation and persistence [9]. Several studies have shown that adherence of hypertensive patients is poor, in both primary and secondary prevention [10–13]. However, it is established that poor adherence to therapy is one of the most important factors to contribute to uncontrolled hypertension. Better adherence to therapy reduces hypertension-related complications [11, 14, 15].

In France, as in many countries, hypertension remains under-diagnosed and under-treated [16], yet the practice of HBPM is increasingly widespread in primary care, even if it is not always applied according to the recommended protocol [16–18]. In contrast to international recommendations [1, 2], the French recommendation, without scientific validation, is to perform 3 measurements in the morning at breakfast and 3 measurements in the evening at bedtime, respecting an interval of one to two minutes between two measures, for 3 days (the rule of 3) [3, 4].

We hypothesized hypertensive patients using HBPM would show better adherence to therapy than those who did not. The primary objective was to determine the relationship between the use of HBPM and adherence in hypertensive patients treated for primary prevention. The secondary objectives were to determine whether patients who used HBPM according to the French guidelines reported better adherence and whether there was a characteristic profile of patients who used HBPM correctly.

## Methods

### Type of study

This was a cross-sectional, comparative study conducted in the Auvergne region between June 1 and November 30, 2016.

#### Study population

Four hundred and fifty general practitioners (GPs) who practised in the region were selected randomly from a list of 1187 obtained from the telephone directory without stratification. A letter of introduction to the study was sent to them by mail, and they were then contacted by telephone. They were then asked to include more than 10 hypertensive patients during a routine visit. These patients received a letter with information to explain the objectives of the study, the conditions of anonymity and confidentiality, together with a self-administered questionnaire containing sociodemographic and medical data, an adherence questionnaire, and a questionnaire on HBPM. The GP had to fill in a table including, for each patient, age, sex, weight, duration of hypertension and current antihypertensive treatment.

The inclusion criteria were: patients aged 18 to 80 years, treated for essential hypertension, in primary prevention with mono-, bi- or triple therapy for more than six months.

The non-inclusion criteria were: secondary hypertension, complicated hypertension treated in secondary prevention, patients who could not read or write or did not speak French, patients judged to have dementia by their GP, patients treated for cancer, diabetes or renal failure (Clearance < 60 mL/min), institutionalized patients, and patients who did not prepare their own treatment.

### Outcome measures

The outcome measures used to meet the primary objective of the study were as follows:

- Medication adherence score, measured using the Girerd self-questionnaire [19, 20]. This questionnaire consists of six questions with binary responses based on factors related to adherence in patients treated for hypertension: 1) Did you omit to take your treatment this morning? 2) Since your last visit, have you run out of treatment? 3) Have you ever taken your treatment later than instructed? 4) Have you ever forgotten to take your treatment? 5) Have you ever decided not to take your treatment because of its side effects? 6) Do you feel that the number of pills you have to take every day is too high? Each positive answer is awarded one point, and the sum of these points gives a score ranging from 0 to 6. If the answer is no to all the questions (i.e., 0 points), adherence is good; if the answer is yes to one or two questions (1 or 2 points), there is minor non-adherence; if 3 or more answers are yes (≥ 3 points), adherence is poor.
- The proportion of patients who reported having an HBPM device.

The criterion used to meet the secondary objective was the proportion of patients who performed HBPM correctly according to the rule of 3. The questionnaire concerning the practice of HBPM had three parts. It evaluates the frequency of measures and the number of measures done each time, the type of device and who suggests its use. We considered that patients who attested practicing 3 measures at the morning and at the evening performed HBPM correctly according to the rule of 3.

### Analysis

Sample size was estimated in order to highlight a relationship between adherence and HBPM monitoring, with adherence expected to be 50% for patients who did not use an HBPM device [21]. To show a relative difference equal to 33% for patients with an HBPM device and making good use of it (10% of sample size), 650 patients were necessary for a two-sided Type I error at 5% and a statistical power of 80% [17, 22].

Statistical analysis was performed using Stata software (version 13, StataCorp, College Station, TX). All tests were two-sided, with a Type I error set at 0.05. Categorical variables were expressed number of subjects and associated percentages, and quantitative variables as mean ± standard deviation or median [interquartile range], according to their statistical distribution. The patients were compared according to their adherence level (good adherence vs minor non-adherence vs poor adherence) on the one hand, and according to their use of HBPM device (HBPM– group, HBPM + group without application of the rule of 3 and HBPM + group with application of the rule of 3) on the other hand. Continuous variables were compared between these groups by ANOVA, or Kruskal–Wallis test if the assumptions of ANOVA were not met. When appropriate (omnibus p-value less than 0.05), post-hoc tests were performed to take into account multiple comparisons: Tukey–Kramer’s test after ANOVA and Dunn’s test after Kruskal–Wallis. Categorical variables were compared between groups using the Chi-squared or Fischer’s exact test. When appropriate, a post-hoc test was used (Marascuilo procedure). In order to determine factors associated with adherence, considered as a three classes variable, a multivariable mixed ordinal regression was carried out (ordered logit), considering covariates according to univariate results (*p* < 0.05) and to clinical relevance. The GP effect was considered as a random effect. Particular attention was paid to the study of multicollinearity and interactions between covariates. Results were expressed as odds ratios (OR) and 95% confidence intervals (CI), and a forest plot was employed to present the results.

### Ethical considerations

The study obtained a favourable ethical advisory opinion from the Ethics Committee of the Clinical Investigation Centres of the Rhone-Alpes-Auvergne interregion (N°IRB 5044). The consent of the patients was obtained in writing.

## Results

### Description of the GP sample

Of the 450 GPs selected at random, 82 (18.2%) included 1046 patients. Because of missing data twenty questionnaire were excluded from the analysis. A total of 1026 patients were included in the analysis (SD1). GPs’ characteristics, compared with the general population of GPs in the Auvergne region in 2016, are presented in Table 1. GPs who practiced in groups were more represented than in the Auvergne region as a whole (71% vs 54% respectively, *p* = 0.003), as well as the clinical tutors (31% vs 16% respectively, *p* = 0.001).

**Table 1:** Characteristics of the 82 general practitioners investigators of the study

	**Sample**	**Overall**^**a**^	***p***
	**(*n* = 82)**	**(*n* = 1263)**	
Male sex	53 (64.6)	755 (59.8)	0.38
Age (years)	51.4 ± 11.0	52.7 ± 11.2	0.31
Department			0.06
Allier	15 (18.3)	312 (24.7)	
Cantal	14 (17.1)	140 (11.1)	
Haute-Loire	7 (8.5)	199 (15.8)	
Puy-de-Dôme	46 (56.1)	612 (48.4)	
Group practice	58 (70.7)	681 (53.9)	0.003
Clinical tutor	25 (30.5)	199 (15.8)	0.001
Urban location^b^	59 (72.0)	878 (69.5)	0.64
HBPM device	60/73 (82.2)	-	-
Self-measurement forms given by GPs	24/73 (32.9)	-	-

Data are presented as frequencies (percentages) or as mean ± standard deviation

*GP* General practitioners, *HBPM* Home blood pressure measurement

^a^General practitioners located in the Auvergne region in 2016 (data from the French national medical council)

^b^Urban location: more than 2500 inhabitants

### Description of the study population

Patient characteristics were compared according to the practice of HBPM (Table 2) and then according to the level of adherence (Table 3). Among the 1026 participants, 521 (50.8%) reported good adherence, 455 (44.3%) minor non-adherence, and 50 (4.9%) non-adherence.

**Table 2:** Description of the 1026 hypertensive patient questioned for the study according to home blood pressure monitoring practice

	**Total** **(*n* = 1026)**	**HBPM − ** **(*n* = 562)**	**HBPM + not using rule of 3** **(*n* = 381)**	**HBPM + using rule of 3** **(*n* = 83)**	**p**
**Patients**					
Age (years)	63.0 ± 10.3	62.4 ± 10.4	63.2 ± 10.3	66.3 ± 9.6	**0.006**^** cd**^
Female sex	588 (57.3)	334 (59.4)	211 (55.4)	43 (51.8)	0.27
Education level	*n* = *994*	*n* = *542*	*n* = *371*	*n* = *81*	
Secondary School	575 (57.8)	332 (61.2)	203 (54.7)	40 (49.4)	
High school	246 (24.8)	131 (24.2)	92 (24.8)	23 (28.4)	0.07
Post-graduate	173 (17.4)	79 (14.6)	76 (20.5)	18 (22.2)	
Occupation	*n* = *960*	*n* = *525*	*n* = *359*	*n* = *76*	
Farmers	23 (2.4)	14 (2.7)	9 (2.5)	0 (0.0)	
Artisans and business people	54 (5.6)	29 (5.5)	21 (5.8)	4 (5.3)	
Managers and higher professions	49 (5.1)	26 (5.0)	21 (5.8)	2 (2.6)	
Intermediate professions	91 (9.5)	51 (9.7)	33 (9.2)	7 (9.2)	0.11
Employees	188 (19.6)	114 (21.7)	69 (19.2)	5 (6.6)	
Workers	52 (5.4)	32 (6.1)	16 (4.5)	4 (5.3)	
Retired	460 (47.9)	232 (44.2)	178 (49.6)	50 (65.8)	
No activity	43 (4.5)	27 (5.1)	12 (3.4)	4 (5.3)	
Smoker	139/1023 (13.6)	95/560 (17.0)	41/381 (10.8)	3/82 (3.7)	**0.001**^**bcd**^
Duration of hypertension	*n* = *999*	*n* = *548*	*n* = *370*	*n* = *81*	
< 5 years	293 (29.3)	156 (28.5)	112 (30.3)	25 (30.9)	
5 to 10 years	282 (28.2)	143 (26.1)	117 (31.6)	22 (27.1)	0.24
> 10 years	424 (42.5)	249 (45.4)	141 (38.1)	34 (42.0)	
Body mass index (kg/m^2^)	27.8 ± 5.1	27.9 ± 5.2	27.8 ± 5.1	26.8 ± 4.5	0.21
SBP (mmHg)	135 ± 13	133 ± 12	136 ± 13	140 ± 15	** < 0.001**^**bcd**^
DBP (mmHg)	77 ± 9	76 ± 9	77 ± 9	79 ± 10	**0.02**^**c**^
Anti-hypertensive medications					
1	704 (68.6)	390 (69.4)	264 (69.3)	50 (60.3)	
2	256 (25.0)	136 (24.2)	94 (24.7)	26 (31.3)	0.55
≥ 3	66 (6.4)	36 (6.4)	23 (6.0)	7 (8.4)	
**General practitioners**					
Age (years)	51.3 ± 10.5	51.0 ± 10.4	51.4 ± 10.8	52.4 ± 9.6	0.50
Male sex	645 (62.9)	361 (64.2)	228 (59.8)	56 (67.5)	0.26
Clinical tutor	412 (40.2)	213 (37.9)	147 (38.6)	52 (62.7)	** < 0.001**^** cd**^
Group practice	771 (75.1)	425 (75.6)	279 (73.2)	67 (80.7)	0.33
Urban location^a^	666 (64.9)	371 (66.0)	257 (67.5)	38 (45.8)	**0.001**^** cd**^

Data are presented as frequencies (column percentages) or as mean ± standard deviation

*DBP* Diastolic blood pressure, *SBP* Systolic blood pressure

^a^Urban location: more than 2500 inhabitants

^**b**^significant difference (*p* < 0.05) between “HBPM − ” and “HBPM + not using rule of 3”;

^**c**^significant difference (*p* < 0.05) between “HBPM − ” and “HBPM + using rule of 3”;

^**d**^significant difference (*p* < 0.05) between “HBPM + not using rule of 3” and “HBPM + using rule of 3”

**Table 3:** Description of the 1026 hypertensive patients questioned for the study according to their medication adherence

	**Good adherence**	**Minor non-adherence**	**Non-adherence**	**p**
**Patients**				
Age (years)	64.3 ± 9.6	61.6 ± 10.6	62.1 ± 13.6	** < 0.001**
Sex				
Male *(n* = *438)*	197 (45.0)	218 (49.8)	23 (5.2)	**0.006**
Female *(n* = *588)*	324 (55.1)	237 (40.3)	27 (4.6)	
Education level				
Secondary School *(n* = *575)*	306 (53.2)	239 (41.6)	30 (5.2)	
High school *(n* = *246)*	123 (50.0)	116 (47.2)	7 (2.8)	**0.01**
Post-graduate *(n* = *173)*	70 (40.5)	90 (52.0)	13 (7.5)	
Occupation				
Farmers (n = 23)	14 (60.9)	9 (39.1)	0 (0.0)	
Artisans and business people *(n* = *54)*	16 (29.6)	32 (59.3)	6 (11.1)	
Managers and higher professions *(n* = *49)*	23 (46.9)	21 (42.9)	5 (10.2)	
Intermediate professions *(n* = *91)*	38 (41.8)	49 (53.8)	4 (4.4)	**0.02**
Employees *(n* = *188)*	98 (52.1)	81 (43.1)	9 (4.8)	
Workers *(n* = *52)*	24 (46.2)	26 (50.0)	2 (3.8)	
Retired *(n* = *460)*	259 (56.3)	182 (39.6)	19 (4.1)	
No activity *(n* = *43)*	24 (55.8)	17 (39.5)	2 (4.7)	
Smoker				
No *(n* = *884)*	466 (52.7)	385 (43.6)	33 (3.7)	** < 0.001**
Yes *(n* = *139)*	53 (38.1)	69 (49.7)	17 (12.2)	
Duration of hypertension				
< 5 years *(n* = *293)*	137 (46.8)	138 (47.1)	18 (6.1)	
5 to 10 years *(n* = *282)*	142 (50.4)	125 (44.3)	15 (5.3)	0.19
> 10 years *(n* = *424)*	230 (54.2)	180 (42.5)	14 (3.3)	
Body mass index (kg/m^2^)	27.7 ± 5.0	27.9 ± 5.4	27.0 ± 5.0	0.44
SBP (mmHg)	135 ± 13	134 ± 12	142 ± 19	** < 0.001**
DBP (mmHg)	76 ± 9	77 ± 9	82 ± 12	** < 0.001**
Anti-hypertensive medications				
1 *(n* = *704)*	372 (52.8)	305 (43.3)	27 (3.9)	
2 *(n* = *256)*	127 (49.6)	113 (44.1)	16 (6.3)	**0.007**
≥ 3 *(n* = *66)*	22 (33.3)	37 (56.1)	7 (10.6)	
**General practitioners**				
Age (years)	51.0 ± 10.6	51.7 ± 10.3	49.8 ± 11.1	0.34
Sex				
Female *(n* = *381)*	190 (49.9)	170 (44.6)	21 (5.5)	0.74
Male *(n* = *645)*	331 (51.3)	285 (44.2)	29 (4.5)	
Clinical tutor				
No *(n* = *614)*	301 (49.0)	283 (46.1)	30 (4.9)	0.37
Yes *(n* = *412)*	220 (53.4)	172 (41.7)	20 (4.9)	
Practice				
Group *(n* = *771)*	388 (50.3)	343 (44.5)	40 (5.2)	0.68
Alone *(n* = *255)*	133 (52.2)	112 (43.9)	10 (3.9)	
Location^a^				
Rural *(n* = *360)*	187 (51.9)	153 (42.5)	20 (5.6)	0.58
Urban *(n* = *666)*	334 (50.2)	302 (45.3)	30 (4.5)	

Data are presented as frequencies (row percentages) or as mean ± standard deviation

*DBP* Diastolic blood pressure, *SBP* Systolic blood pressure

^a^Urban location: more than 2500 inhabitants; rural location: less than 2500 inhabitants

### Profile of patients using HBPM according to the rule of 3

The results of the bivariate analysis (Table 2) showed that patients using HBPM according to the rule of 3 were significantly older (66.3 ± 9.6 years vs 63.2 ± 10.3 years for HBPM + without rule of 3 and 62.4 ± 10.4 years for HBPM-, *p* = 0.006), and were more likely to be non-smokers (*p* = 0.001).

### Adherence by HBPM practice

**Fig. 1:**
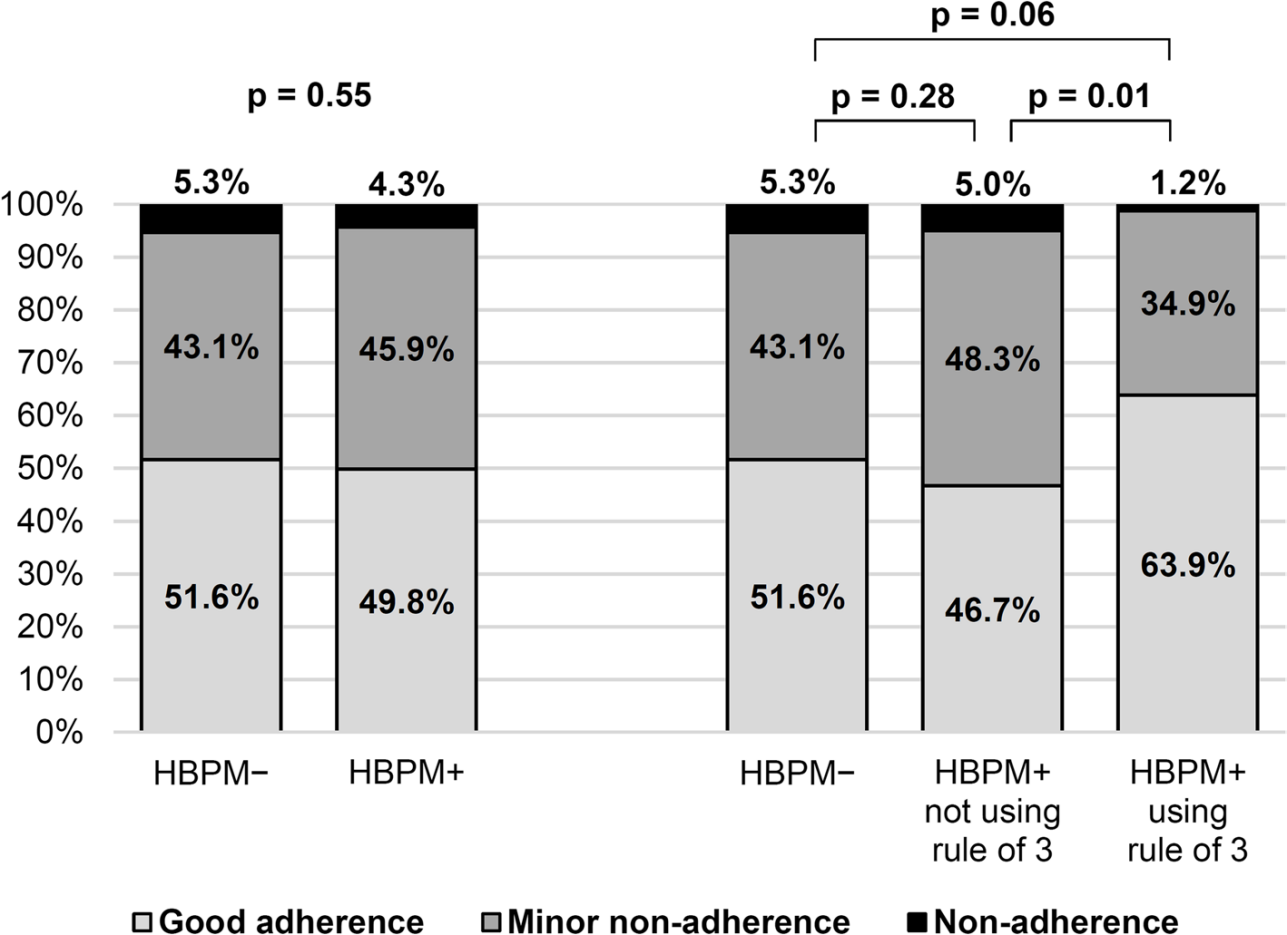
Reported adherence of 1026 hypertensive patients according to their practice of HBPM (Bivariate analysis)

A total of 464 patients (45.2%) reported having an HBPM device (HBPM + group). Of these, 83 (17.9%) were aware of the recommended rule of 3. There was no significant difference in adherence between those in the HBPM + group and those who reported not having one (HBPM– group) (*p* = 0.55) (Fig. 1). In univariate analysis there was a significant difference (*p* = 0.04) among the three groups (HBPM– group, HBPM + group without application of the rule of 3 and HBPM + group with application of the rule of 3). Groups were then compared in pairs (Fig. 1). There was no significant difference in adherence between patients in the HBPM– group and: 1) those in the HBPM + group who doesn’t use the rule of 3 (*p* = 0.28), 2) those in the HBPM + group using the rule of 3 (*p* = 0.06). The self-reported adherence of patients using HBPM according to the rule of 3 was better than that of patients not using HBPM according to the rule of 3 (*p* = 0.01). Patients in the HBPM + using rule of 3 group had better adherence than those in the other two groups combined (*p* = 0.03).

### Multivariable analysis

**Fig. 2:**
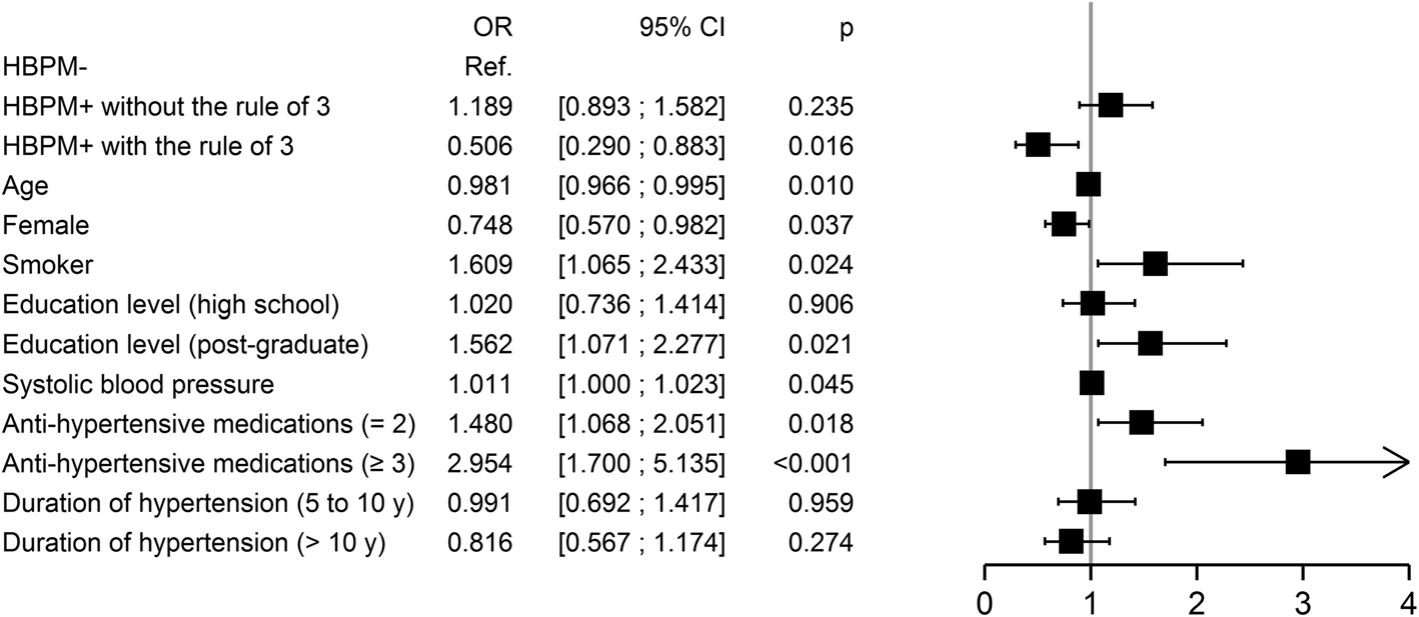
Reported adherence of 1026 hypertensive patients according to the practice of HBPM and adjusted on patient factors. (Multivariable analysis)

After adjusting for age, sex, smoking status, education level, systolic blood pressure, duration of hypertension, and number of medications (Fig. 2), the results confirm those of the bivariate analysis. Patients who practiced HBPM using the rule of 3 reported better adherence to therapy than those in the HBPM– group (OR: 0.51, 95% CI: 0.29–0.88, *p* = 0.02); Patients in the HBPM + group who did not use the rule of 3 did not report better adherence than those who did not have a device(OR: 1.19, 95% CI: 0.89–1.58, *p* = 0.24). Within the HBPM + group, patients who measured according to the rule of 3 reported better adherence than those who did not (*p* = 0.003).

By integrating each factor independently, the results of multivariable analysis identified factors associated with good adherence: older age, female sex, non-smoking, low systolic blood pressure, given treatment as monotherapy, and education level under post graduate.

## Discussion

### Main results

In our survey of 1026 participants, one out of two reported good adherence. This level of adherence is comparable with recent studies that evaluated adherence in primary prevention in cardiovascular disease [10]. We chose to include hypertensive patients treated in primary prevention to evaluate adherence at a stage when hypertension is only a risk factor, without cardiovascular complications. Patients who reported good adherence were older, female, non-smokers, treated with monotherapy, and not highly educated. A total of 464 (45.2%) participants reported owning an HBPM device. Patients who used HBPM according to the rule of 3 reported better adherence than those who did not own a device and those who had a device but did not use it according to the recommended rule.

Older patients were more likely to use HBPM according to the recommended rule, probably reflecting a greater concern for risk factor control with age. On the contrary, patients who smoked and use HBPM less, may be less concerned about their health. Patients using HBPM according to the recommended rule were more likely to have a clinical tutor, working in a rural setting.

### Comparison with literature

Primary care studies show that hypertensive patients are increasingly owning an HBPM device [22–25]. Nevertheless, too few patients know the correct rule of for its use, although in our study the rate was higher in comparison with a similar French study in 2012 (12% versus 2%) [22]. One explanation is the self-reported survey done at a single time. It exposes to the risk of social desirability bias and reporting bias. It consists in responding what is expected to seem to be a “good” patient. To minimize this bias, we informed patients about anonymity and also recruited a high number of participants to mix many points of view. Also, we did not clearly mention the rule of 3 in the survey but only asked questions about its term and condition so that patients were not influenced to answer the question.

The results of studies to evaluate the effect of HBPM on adherence are controversial, with results varying between no effect [26, 27] and a definite benefit [28]. For two reviews of the literature, the benefit was moderate [6, 7]. These reviews included interventional studies with mixed results. The methods used were heterogeneous, particularly for assessing adherence. We did not find any study evaluating the effects of HBPM on adherence with the rules recommended in the various national guidelines. The assessment of the association between adherence and HBPM was global, with no distinction between good and bad users. Multivariable analyses showed that lower systolic blood pressure was a factor associated with good adherence. It supposes that patients well controlled by anti-hypertensive treatment have better adherence. It is unsure these patients are all practicing monitoring. The link between HBPM alone and better control of blood pressure is not sure. A meta-analysis from Tucker [5] showed that practice of HBPM alone does not improve control of hypertension. It became significant if HBPM is associated with other interventions such as self-titration of medication and lifestyle counselling by pharmacist or nurse. This enhances the result of our study. The practice of HBPM has to be accompanied by professional, and be performed rigorously for a better adherence and so a better control of hypertension. Adherence is influenced by sociocultural and psycho-behavioral factors specific to patients, and the quality of the doctor–patient relationship [29, 30]. It is likely that participants in our survey who used the HBPM correctly received better information from their GP, thus developing self-care competencies [31]. This is consistent with trials that have evaluated the impact of HBPM on reducing BP. Hypertension decreases more when the use of HBPM is combined with other interventions such as telemonitoring, lifestyle counselling and therapeutic education [5, 32, 33].

### Strengths and limitations

This study was geared to the assessment of the end points. The recruitment method used should allow reliable generalization of the results to all hypertensive patients in the region. However, the number of GPs who actually included participants was quite small, with an overrepresentation of group practice GPs and clinical tutors. Apart from these criteria, the sample of GPs in the study is quite similar to the overall GP population in the region. It is likely that it was the most motivated GPs who agreed to participate in the study, with practices likely to be fairly similar, and this may have minimized differences among the groups. Another limitation was that GPs did not select patients consecutively because of omission, lack of time, or because they chose to include the most cooperative patients.

The declarative nature of a self-administered survey should also be taken into consideration. Adherence was assessed only by a self-administered questionnaire. It is accepted that this type of measure is a good estimate of adherence [34], although a combination of methods is preferable to measure the components of adherence: initiation, implementation and persistence [35]. Patients in the study were required not to have diabetes or serious medical conditions. However, psychosocial factors, depressive symptoms or comorbidities could be associated with poorer adherence.

## Conclusions

In our study, approximately one in two participants reported owning an HBPM device. Only 18% of them knew the rules regarding its use for the monitoring of hypertension. Their reported adherence was better than that of patients who did not have a device and that of patients who had one but did not use it properly. This reinforces the idea that the practice of HBPM must be accompanied by the information and therapeutic education necessary for the proper management of hypertension.

## Supplementary Information

Additional file 1. (TIF 37 kb)

## Data Availability

The datasets used and/or analyzed during the current study are available from the corresponding author on reasonable request.

## Abbreviations

BP: Blood Pressure
ESH: European Society of Hypertension
GP: General Practitioner
HBPM: Home Blood Pressure Monitoring
