# Supplementary figures and images for: Home blood pressure monitoring and adherence in patients with hypertension on primary prevention treatment: a survey of 1026 patients in general medicine in the Auvergne region
Source: BMC Prim Care. 2022 May 26;23:131. doi: 10.1186/s12875-022-01725-8 (PMC9137195; doi:10.1186/s12875-022-01725-8)

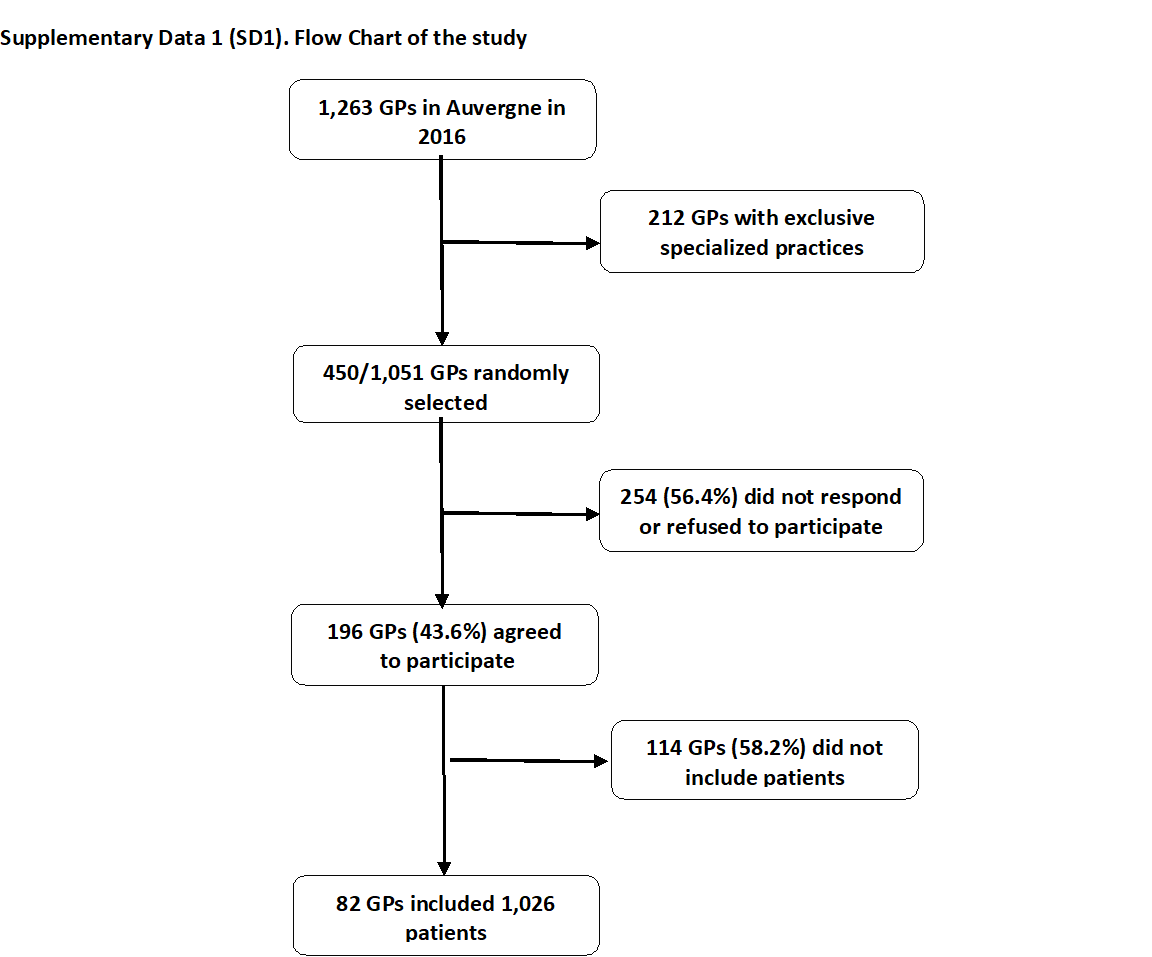

Supplement: Supplementary file 1 — Additional file 1. (TIF 37 kb) [file 12875_2022_1725_MOESM1_ESM.tif]
